# Comparative Proteomics Analysis of Exosomes Identifies Key Pathways and Protein Markers Related to Breast Cancer Metastasis

**DOI:** 10.3390/ijms24044033

**Published:** 2023-02-17

**Authors:** Shichen Shen, Chengjian Tu, He Shen, Jun Li, Costa Frangou, Jianmin Zhang, Jun Qu

**Affiliations:** 1 Department of Pharmaceutical Sciences, University at Buffalo, Buffalo, NY 14214, USA; 2 New York State Center of Excellence in Bioinformatics and Life Sciences, Buffalo, NY 14203, USA; 3 Department of Cancer Genetics and Genomics, Roswell Park Comprehensive Cancer Center, Buffalo, NY 14263, USA; 4 Department of Molecular and Cellular Biology, Roswell Park Comprehensive Cancer Center, Buffalo, NY 14263, USA

**Keywords:** exosomes, breast cancer, metastasis, quantitative proteomics, label-free quantification, IonStar

## Abstract

Proteomics analysis of circulating exosomes derived from cancer cells represents a promising approach to the elucidation of cell–cell communication and the discovery of putative biomarker candidates for cancer diagnosis and treatment. Nonetheless, the proteome of exosomes derived from cell lines with different metastatic capabilities still warrants further investigation. Here, we present a comprehensive quantitative proteomics investigation of exosomes isolated from immortalized mammary epithelial cells and matched tumor lines with different metastatic potentials in an attempt to discover exosome markers specific to breast cancer (BC) metastasis. A total of 2135 unique proteins were quantified with a high confidence level from 20 isolated exosome samples, including 94 of the TOP 100 exosome markers archived by ExoCarta. Moreover, 348 altered proteins were observed, among which several metastasis-specific markers, including cathepsin W (CATW), magnesium transporter MRS2 (MRS2), syntenin-2 (SDCB2), reticulon-4 (RTN), and UV excision repair protein RAD23 homolog (RAD23B), were also identified. Notably, the abundance of these metastasis-specific markers corresponds well with the overall survival of BC patients in clinical settings. Together, these data provide a valuable dataset for BC exosome proteomics investigation and prominently facilitate the elucidation of the molecular mechanisms underlying primary tumor development and progression.

## 1. Introduction

Breast cancer (BC) is the most frequently diagnosed cancer and the second leading cause of cancer-related mortality among women in the US. According to the American Cancer Society, a total of 281,550 and 49,290 newly diagnosed cases of invasive and non-invasive (i.e., in situ) BC, respectively, were expected, and approximately 43,600 deaths were estimated among US women in 2021 [1]. Surgical operations to remove cancerous tissues, combined with other therapeutic approaches, such as chemotherapy, are an effective strategy with which to eliminate primary tumors and decrease the chances of potential recurrence [2]. However, the high metastasis propensity of BC regarding key organs and tissues (e.g., the lymph nodes, bones, brain, liver, lungs, and kidneys) renders the prognosis for BC patients extremely poor and is responsible for the vast majority of BC-related deaths [3]. Therefore, further research to better understand the molecular mechanisms underlying the development and progression of metastatic BC, as well as the discovery of circulatory biomarkers to establish more sophisticated measures for the early diagnosis of BC patients who are at a higher risk of metastasis, will be of profound significance for the optimization of treatment regimens and improvement in the BC patient survival rate.

Exosomes are one subtype of extracellular vesicles (EV) secreted by various cell types into the extracellular space, and they contain several categories of biomolecules (e.g., proteins, DNAs, RNAs, and lipids) [4]. These 40–100 nm cell-derived nanoparticles are present in multiple types of biological fluids and are actively involved in cell–cell communication and signal transduction, which are key to numerous physiological and pathological processes. Accumulating evidence has also shown that cancer-cell-derived exosomes act as critical mediators of tumor stroma microenvironment remodeling and play essential roles in the progression, invasion, and metastasis of a number of cancers, including BC [5,6,7]. Previous studies have reported that exosomes derived from BC contain a list of well-studied cancer-associated molecules [7]; additional research has also demonstrated the potential application of exosomes in the discovery of biomarkers targeting BC diagnosis and treatment [8,9,10,11].

Discovery-based investigations of exosome protein composition using state-of-the-art quantitative proteomics approaches have prominently contributed to research efforts in both regards [12,13,14,15]. Nonetheless, while these studies have demonstrated the feasibility of applying quantitative proteomics to investigate exosomes and the great potential of BC-derived exosomes for biomarker discovery and mechanism studies, most of them have employed metastatic and non-metastatic cell lines of different origins or biofluids collected from individuals with completely different clinical profiles. Few studies have investigated how exosomes from the same mammary epithelial cell lineage differ from one another during the transition from normal to tumorigenic/metastatic states.

Here, we present a comprehensive quantitative proteomics investigation of exosomes from five BC cell lines with different metastatic capabilities and metastasis sites, all of which were derived from the same parental mammary epithelial cell line, EpH4-Ev. Exosomes were isolated using a centrifugation-based method and analyzed via a unique MS1 ion current-based quantitative proteomics pipeline, IonStar. As demonstrated by our previous studies, IonStar provides reliable protein quantification, with excellent accuracy/precision and extremely low missing data and false biomarker discovery rates, and its performance has surpassed several prevalent methods, such as MaxQuant and SWATH-MS, especially for low-abundance proteins [16,17,18]. Proteins were significantly changed in each cell line, along with key biological functions and signaling pathways that were dysregulated in the tumorigenic/metastatic cell lines, as was determined by comparing each cell line to the parental cell line and applying a suite of informatics approaches. Furthermore, a survival analysis was performed to correlate the changed proteins specific to the three metastatic cell lines with invasive BC patient survival to identify potential exosome protein markers for BC diagnosis and prognosis.

## 2. Results

### 2.1. Reliable Proteomics Quantification of BC-Cell-Derived Exosomes

**Figure 1:**
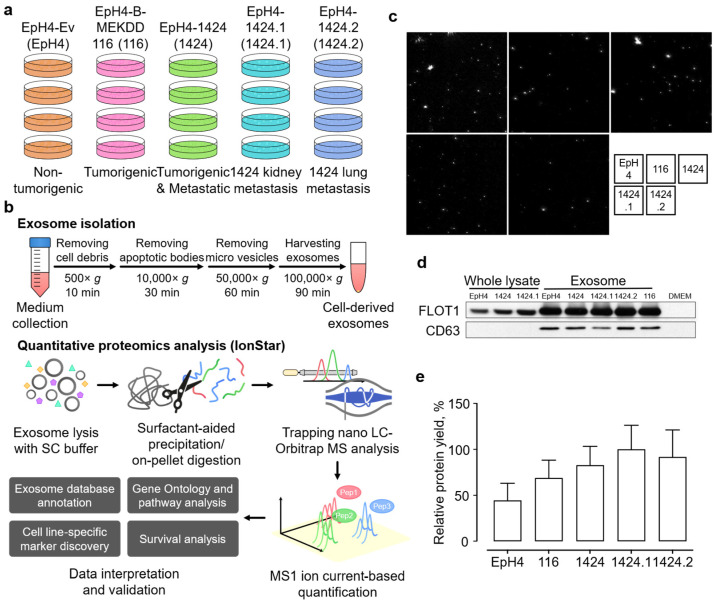
Quantitative proteomics investigation of exosomes isolated from primary and derived EpH4-Ev cell lines with different tumorigenic and metastatic capabilities. (**a**) Specification of samples in this study; (**b**) the scheme for exosome sample preparation and proteomic experimental procedures; (**c**) images of exosomes isolated from the five cell lines; (**d**) Western blot assay of exosome markers FLOT1 and CD63 in whole-cell lysate and isolated exosome samples; (**e**) relative protein yield from exosome samples among the five cell lines.

The overall study scheme is presented in Figure 1a,b. EpH4-Ev, a non-tumorigenic mouse mammary epithelial cell line, was selected as the baseline control for proteomics comparison. In addition, four derived tumorigenic cell lines were also employed in the study, including EpH4-Ev, EpH4-B-MEKDD 116, and EpH4-1424 (primary tumor cells) and EpH4-1424.1 (kidney metastasis cells) and EpH4-1424.2 (lung metastasis cells) [19]. These cell lines have been frequently adopted in the investigation of the epithelial-to-mesenchymal transition (EMT), as well as metastasis [20]. Exosomes were isolated via an efficient and robust multi-step centrifugation procedure and subjected to a surfactant cocktail (SC)-aided extraction/precipitation/on-pellet digestion (SEPOD) protocol for exhaustive and reproducible protein digestion [21]. Imaging results showed that a large proportion of isolated exosomes had a diameter of ~100 mm and there were no significant differences among the five cell lines (Figure 1c and Figure S1). To further evaluate the purity of the isolated exosomes, we performed an immunoblot analysis for two well-established exosome markers, Flotillin-1 (FLOT1) and CD63 antigen (CD63). As expected, we detected a significant enrichment of FLOT1 in the exosome fractions as compared to whole lysate, while CD63 was exclusively detected in the exosome fractions (Figure 1d). These results together suggest the high efficiency and selectivity of the exosome isolation procedures. Notably, exosomes from the four tumorigenic cell lines yielded higher amounts of protein as compared with the non-tumorigenic EpH4 control (Figure 1e).

**Figure 2:**
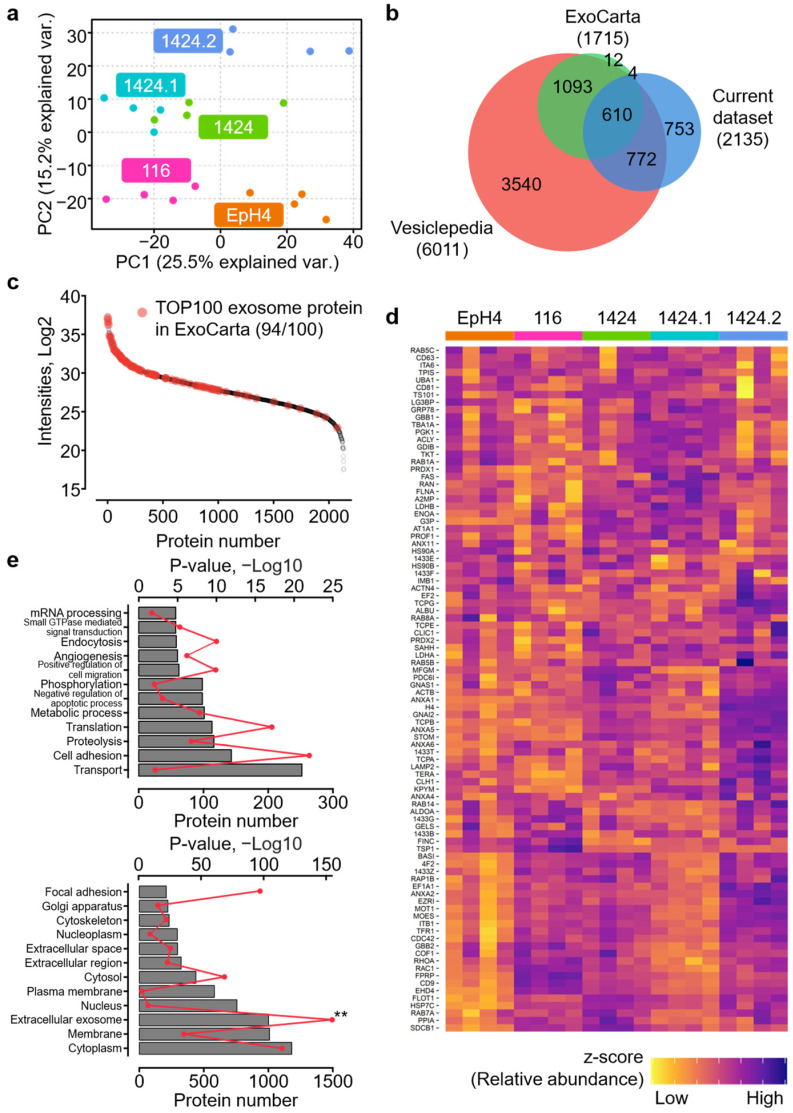
Proteomics characterization of the isolated exosomes. (**a**) Principal component analysis (PCA) of the proteomic profiles of the five cell lines; (**b**) Venn diagram showing the overlap between the proteins quantified in the current dataset and those archived in two extracellular vesicle databases, Vesiclepedia and ExoCarta; (**c**) ranked MS intensities of the quantified proteins. Red dots denote TOP100 exosome proteins in theExoCarta database; (**d**) normalized abundance of the 94 ExoCarta TOP100 exosome proteins quantified in this dataset; (**e**) gene ontology (GO) biological process (BP) and cellular component (CC) terms enriched from the exosome proteome. ** *p*-value = 0 for the extracellular exosome GO CC term.

The derived peptides were analyzed by using a trapping nano liquid chromatography (LC)–Orbitrap mass spectrometry (MS) system, which features a selective peptide delivery strategy and long-column, long-gradient nano LC separation to maximize the sensitivity of the LC–MS [16]. LC–MS raw files were analyzed by using the UHR-IonStar data processing pipeline in an in-house-developed R-shiny APP to generate the quantification results. The LC–MS raw files are available on the ProteomeXchange Consortium with the dataset identifier PXD038553. For the five cell lines, an average of 1017 ± 53 proteins were identified at 1% protein/peptide false discovery rate (FDR) and ≥2 unique peptides per proteins (Figure S2a); the highest number of proteins was observed in the 1424 cell line (1080 ± 10), while the lowest number of proteins was observed in the 1424.1 cell line (955 ± 12). Combining all samples together, a total of 2135 proteins were quantified in the 20 exosome samples, with mean intra-group coefficient of variation (CV) levels ranging from 13.4% to 18.0% (Figure S2b). The list of proteins quantified and their MS quantitative values in each sample can be found in Table S1. The intra-group CV level of the LC–MS technical replicates was ~8%, suggesting excellent technical reproducibility for both the experimental procedures and the data processing pipeline. Principal component analysis (PCA) revealed cell-line-specific proteomic profiles among the five cell lines (Figure 2a); in particular, the three tumor-derived 1424 cell lines exhibited more unique signature patterns when compared to the non-tumorigenic EpH4 and tumorigenic EpH4-Ev cell lines. Moreover, the proteomic profile of the 1424.1 cell line, derived from kidney metastasis of 1424, appeared to be more similar to the primary tumor 1424 cell line than the 1424.2 cell line derived from lung metastasis of 1424. These results indicate that exosome-mediated cell–cell communication likely involves the activation of alternative biological functions and signaling pathways in BC lung metastasis.

Among the proteins quantified, 1382 (64.7%) were archived in the *Mus musculus* section of two publicly available extracellular vesicle databases, Vesiclepedia [22] and ExoCarta [23] (protein and/or mRNA). In addition, 589 out of the 753 remaining proteins were archived in the *Homo sapiens* section. This suggests that the majority of proteins quantified in the current study (1971, 92.3%) are well documented in the extracellular fraction, proving the value of this dataset (Figure 2b). Notably, 94 out of the TOP100 proteins in the ExoCarta database were also quantified with high confidence in this dataset, which spanned six orders of intensity (Figure 2c and Table S2). The abundance of these 94 representative exosome protein markers also exhibited cell line-specific patterns among the five cell lines investigated (Figure 2d). Furthermore, seven canonical exosome markers, including three tetraspanins (CD9, CD63, and CD81) and four multivesicular body (MVB)-associated proteins (PDC6I, TS101, CLH1, and FLOT1) were also quantified with high reproducibility and exhibited cell-line-specific patterns (Figure S2c).

To obtain deeper insights into the biological functions and cellular localizations of the proteins characterized in the isolated exosomes, a gene ontology (GO) analysis was performed. A significant enrichment of proteins in the membrane and extracellular exosome GO cellular component (CC) terms, both with >1000 proteins, was observed (Figure 2e, lower panel). Other major GO CC terms that were enriched include the cytoplasm, nucleus, extracellular region, Golgi apparatus, and cytoskeleton. On the other hand, a list of biological process (BP) terms were also enriched with high statistical significance, e.g., transport, cell adhesion, proteolysis, translation, metabolic process, negative regulation of apoptotic process, and phosphorylation (Figure 2e, upper panel). Moreover, associations between the exosome proteins quantified in this dataset and human diseases were identified using DisGeNet [24] (Table S3). Intriguingly, the most significantly enriched terms were malignant neoplasm of breast (187 proteins), breast carcinoma (111 proteins), mammary neoplasms (108 proteins), and neoplasm metastasis (54 proteins). These results together suggest that the proteins quantified in this current dataset were genuinely localized in the exosome and membrane fractions and were functionally relevant to the tumorigenic and metastatic properties of the 116 and 1424 cell lines.

### 2.2. Characterization of Proteomic Discrepancies among BC Cell Lines

**Figure 3:**
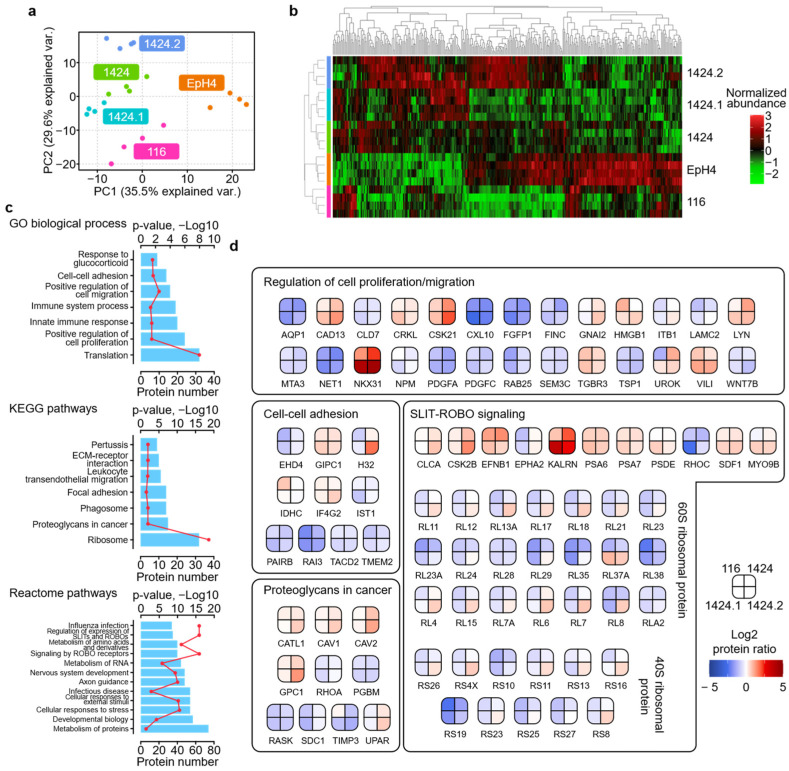
Analysis of proteins with significantly different abundances between the five cell lines. (**a**) Principal component analysis (PCA) of 348 altered proteins; (**b**) heatmap of altered proteins with unsupervised hierarchical clustering; (**c**) GO BP, KEGG, and Reactome pathway terms for which altered proteins were enriched; (**d**) altered proteins enriched in GO BP terms and KEGG/Reactome pathways relevant to cell migration and metastasis.

To better assess the alterations between the exosome proteomes of the five cell lines, the next step was to subset a group of proteins with significantly different abundances between tumorigenic cell lines with and without metastatic capabilities (i.e., tumor) and the non-tumorigenic cell line (i.e., control). A one-way ANOVA was performed on all proteins quantified, and to further enhance the sensitivity and specificity of this step, protein ratio thresholds of > 1.5 or < 0.67 were applied in addition to the ANOVA *p*-value threshold of < 0.05. As a result, a total of 348 proteins were determined to be significantly different in at least one set of the tumor/control comparison (Table S4). The lists TOP20 altered proteins with the highest fold change values in each set of tumor/control comparison are enclosed in Table S5. The PCA results of these altered proteins showed more distinct separation among the three categories of cell lines: the non-tumorigenic EpH4 cell line; the tumorigenic and non-metastatic 116 cell line; and the tumorigenic and metastatic 1424 cell lines (Figure 3a). Remarkably, the 1424.1 cell line appeared to be more similar to the primary tumor 1424 cell line than the 1424.2 cell line (lung metastasis), consistent with the PCA results using all quantified proteins. Unsupervised hierarchical clustering also revealed cell-line-specific patterns regarding altered proteins among the three categories of cell lines (Figure 3b). Interestingly, a sizable cluster of proteins showed higher abundance in the 1424.2 cell line as compared to 1424 and 1424.1 cell lines, which corresponds with the PCA results. These results substantiated the hypothesis that the exosome protein compositions of the five cell lines were significantly different and likely correlated with the tumorigenic and metastatic properties of each cell line. 

GO and pathway analysis, including KEGG [25] and Reactome pathway analysis (RPA) [26], was then performed based on the 348 altered proteins to acquire a deeper understanding of how biological functions and pathways were dysregulated in the tumorigenic and metastatic cell lines. The TOP10 enriched GO CC terms were membrane (186), extracellular exosome (170), cytoplasm (152), plasma membrane (116), extracellular space (60), extracellular region (59), cytosol (52), focal adhesion (49), intracellular (44), and cell surface (42), suggesting that the majority of proteins with significantly different abundances were genuinely localized in the exosome fractions of the cells. The number of altered proteins and corresponding *p*-values for GO BP terms, KEGG pathways, and the TOP12 enriched Reactome pathways are shown in Figure 3c. We found that the enriched GO BP terms and KEGG/Reactome pathways can be assigned to four sub-categories based on their functional similarities, which encompass (1) the activation of innate immunity: innate immune response (20), immune system process (19), phagosome (14), pertussis (9), infectious disease (54), and influenza infection (34); (2) the regulation of cell adhesion and migration: positive regulation of cell migration (16), cell–cell adhesion (14), focal adhesion (14), ECM-receptor interaction (10), axon guidance (48), signaling by ROBO receptors (40), and regulation of expression of SLIT and ROBOs (35); (3) cancer-related signaling: positive regulation of cell proliferation (24), response to glucocorticoid (9), and proteoglycans in cancer (15); and (4) cell metabolism: translation (32), metabolism of proteins (74), metabolism of RNA (44), and metabolism of amino acids and derivatives (40). In addition, 25 and 13 altered proteins were enriched in the DisGeNet terms breast carcinoma and metastasis, respectively (Table S6).

Furthermore, we examined altered proteins involved in key GO terms and KEGG/Reactome pathways relevant to cell migration and metastasis (Figure 3d), including regulation of cell proliferation/migration, cell–cell adhesion, proteoglycans in cancer, and SLIT-ROBO signaling. Among these proteins, we also found a list of previously reported protein markers for the metastasis of various types of cancers, which showed increased abundance in the three metastatic 1424 cell lines as opposed to the EpH4-B-MEKDD 116 cell line, including tyrosine-protein kinase Lyn (LYN), homeobox protein Nkx-3.1 (NKX31), casein kinase II subunit alpha (CSK21), caveolin-1 and -2 (CAV1 and CAV2), kalirin (KALRN), glypican-1 (GPC1) and villin-1 (VILI). Moreover, a large number of 60s and 40s ribosomal protein family members were enriched; while these proteins showed a predominantly decreased abundance in all four tumorigenic cell lines as compared to the non-tumorigenic EpH4 control cell line, more than half of them exhibited an increased abundance in the 1424.2 cell line, implicating the elevated secretion of ribosomal proteins, as an exosome cargo was specific to the 1424.2 cells and may participate in the metastasis process in BC. The functional relevance of the four sub-categories described above, as well as the altered proteins playing key regulatory roles in BC tumorigenesis and metastasis, are further elaborated in the Discussion section. 

### 2.3. Identification of Metastasis-Specific Proteomic Signatures

**Figure 4:**
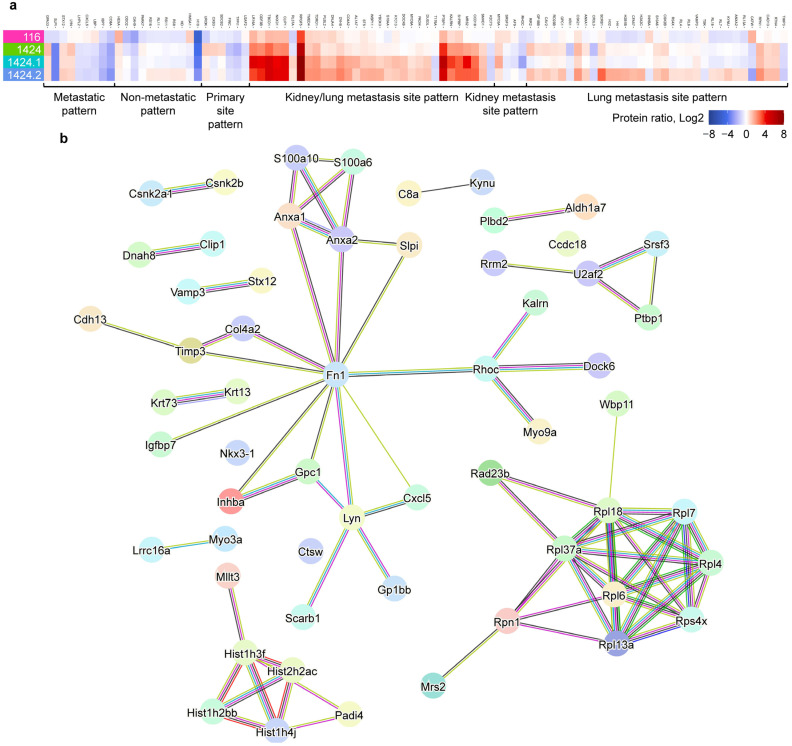
Determination of metastatic-specific altered proteins. (**a**) Classification of metastasis-specific altered proteins by their abundance profiles in the metastatic and non-metastatic BC cell lines. (**b**) Protein–protein interaction (PPI) network of the 83 metastasis-specific proteins, generated by STRINGDB.

One of the ultimate goals of the current study was to identify putative protein markers specific to BC metastasis. In order to achieve this goal, we first dissected the altered proteins to select those better representing metastasis-specific protein changes. An additional set of protein ratios was calculated between the three metastatic cell lines (i.e., 1424, 1424.1, and 1424.2) and the non-metastatic cell line (i.e., 116), and metastasis-specific proteins were defined as altered proteins with a protein ratio of > 1.5 or < 0.67 in at least one out of the three sets of comparisons between metastatic and non-metastatic cell lines. Under the given criteria, a total of 94 altered proteins were selected (Table S7), and the lists of TOP20 altered proteins with the highest fold change values in each set of tumor/control comparison are enclosed in Table S8. Hierarchical clustering was performed to further classify these metastasis-specific proteins into the following patterns (Figure 4a): (1) metastatic pattern (9): more significant in all metastatic cell lines; (2) non-metastatic pattern (11): more significant in the non-metastatic 116 cell line; (3) primary site pattern (6): more significant in the 1424 cell line (primary site); (4) kidney/lung metastasis site pattern (31): more significant in the 1424.1 and 1424.2 cell lines (metastasis sties); (5) kidney metastasis site pattern (4): more significant in the 1424.1 cell line; and (6) lung metastasis site pattern (33): more significant in the 1424.2 cell line. Metastasis-specific proteins are defined as altered proteins with more significantly changed abundances in the metastatic cell lines; therefore, we excluded the eleven altered proteins from Pattern 2. Intriguingly, a large proportion of the metastasis-specific proteins were classified as kidney and/or lung metastasis-specific (68 out of 83), suggesting the potential role of exosome-based signaling transduction in the metastasis of BC from primary to secondary sites. Specifically, 31 metastasis-specific proteins were determined to be lung metastasis site specific, implying that a unique set of signaling pathways may be involved in the lung metastasis of BC. STRINGDB analysis [27] was then performed on the 83 metastasis-specific proteins to construct the protein–protein interaction (PPI) network and the corresponding functional nodes (Figure 4b), and it was evident that several PPI sub-networks were identified, implicating that metastasis-specific proteins might be functionally co-regulated during BC cell metastasis.

**Figure 5:**
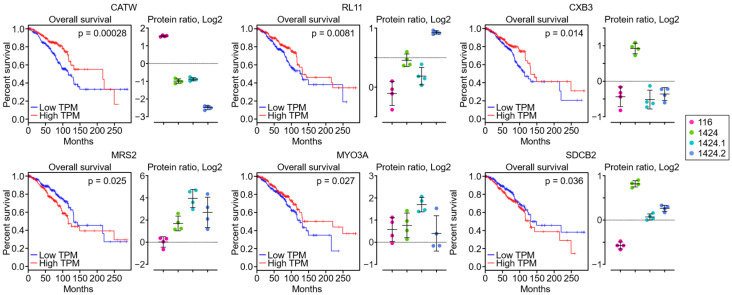
Survival plots and corresponding protein exosome abundances of six metastatic-specific altered proteins with *p* < 0.05 in the survival analysis.

Furthermore, a survival analysis was performed using Gene Expression Profiling Interactive Analysis (GEPIA) on the 83 metastasis-specific proteins to associate their abundances as exosome cargos with the survival lengths of invasive BC patients. As a result, six out of the eighty-three metastasis-specific proteins were statistically significant (i.e., *p*-value < 0.05), including cathepsin W (CATW or CTSW), 60S ribosomal protein L11 (RL11), gap junction beta-3 protein (CXB3), magnesium transporter MRS2 (MRS2), myosin-IIIa (MYO3A), and syntenin-2 (SDCB2). Survival plots and protein abundances are shown in Figure 5. Among these six metastasis-specific proteins, the abundances of CATW, MRS2, and SDCB2 in the metastatic cell lines corresponded well with the clinical profiles of invasive BC patients. Specifically, the lower exosome abundance of CATW was correlated with lower gene expression and worse survival rates in invasive BC patients; on the other hand, the higher exosome abundance of MRS2 and SDCB2 was correlated with higher gene expression and worse survival rates in invasive BC patients. Such correlational trends were not observed for RL11, MYO3A, or SDCB2. In addition, although they did not meet the *p*-value < 0.05 threshold, reticulon-4 (RTN, *p*-value = 0.064) and UV excision repair protein RAD23 homolog (RAD23B, *p*-value = 0.093) also showed negative correlations with invasive BC patient survival, where the higher exosome abundances of RTN and RAD23B were correlated with higher gene expression and worse survival rates in invasive BC patients (Figure S3). The biological and clinical relevance of these putative BC metastasis markers are discussed below.

## 3. Discussion

### 3.1. Comparative Characterization of BC Cell Exosome Proteome

In the past decade, evidence has been collected revealing the pivotal role exosomes play in cancer initiation, proliferation, metastasis, and chemotherapy resistance [28]. Extensive investigations of exosome protein cargos via quantitative proteomics approaches have also been conducted, facilitating the discovery of putative protein biomarkers for cancer diagnosis and prognosis, as well as the elucidation of the molecular mechanisms underlying the etiology of various cancers. In the context of BC, endeavors have also been made to demonstrate that BC exosome proteomic cargos actively participate in the etiological process of BC and have great potential in practical applications, such as BC subtyping and site prediction of BC metastasis [12,13,14,15]. In the current study, exosomes derived from the same mammary epithelial cell lineage (EpH4-Ev) with distinct tumorigenic and metastatic properties were isolated via centrifugation and analyzed using LC–MS-based proteomics, which eliminated baseline variability due to differences between cell lineages and enabled a more unbiased investigation of how the profiles of exosome protein cargos shift during the transition from non-tumorigenic epithelium to BC tumor cells with metastatic potentials. A total of 2135 proteins were quantified with no protein-level missing data in the five cell lines (N = 20 in total) using the IonStar approach, among which > 92% were documented in the Vesiclepedia and ExoCarta databases, reflecting the extraordinary efficiency of the exosome isolation procedures and the high fidelity of the data to the protein cargos being secreted from these mammary epithelium and BC tumor cell lines. In addition, 182 proteins in the current dataset were not archived in either extracellular vesicle database, but no significant differences in MS intensities were observed between archived and non-archived proteins (Figure S4), suggesting the possibility that these non-archived proteins could be an integral part of the exosome proteome as well. GO analysis has revealed that a large proportion of the non-archived proteins were involved in transcription-related processes, cellular amino acid metabolic process, and protein post-translational modification.

Proteins with significantly different abundance levels among the five cell lines were determined via one-way ANOVA and protein ratio cutoff thresholds, leading to the identification of 348 altered proteins. Combining hierarchical clustering, PCA, and Pearson correlation analysis, we found that these altered proteins were distinctive for each cell line, implying that the exosome proteomic cargos transported by the tumorigenic and metastatic cell lines may be actively participating in the etiological processes of BC, including but not limited to cancer proliferation, survival, invasion, and metastasis. To further delineate the functional relevance of the altered proteins, we conducted GO and pathway analysis (KEGG and RPA) and summarized four sub-categories of GO BP terms and KEGG/RPA pathways based on the enrichment results. 

Tumor-derived exosomes have been considered as one of the critical components in preparing for the pre-metastatic niche in breast cancer [29]. Our analysis of the altered proteins identified the enrichment of ribosomal proteins and proteoglycan proteins in lung and kidney metastasis cells, such as GPC1, integrin beta-1 (ITB1), and syndecan-1 (SDC1). GPC1-enriched tumor exosomes contain mutant KRAS mRNA and have been indicated as a reliable biomarker for the detection of early pancreatic cancer [30]. Notably, exosomal integrins (ITGs) have been shown to direct the organ-specific colonization of tumor cells, thus initiating pre-metastatic niche formation [31]. Further analysis of our newly identified exosomal proteoglycan proteins will help to develop new breast cancer metastasis biomarkers.

### 3.2. Identification of Potential Exosome Markers for BC Metastasis and Patient Survival

In order to identify putative protein markers specific to BC metastasis, altered proteins with a > 50% fold change between the three tumorigenic and metastatic cell lines and the tumorigenic and non-metastatic cell line were subset and classified by hierarchical clustering. A total of 83 metastasis-specific proteins were selected, and with a survival analysis, we found that among the six metastasis-specific proteins for which there was a significant correlation (i.e., *p* < 0.05) between gene expression levels and overall survival length in invasive BC patients, the exosome abundance of three proteins in the metastatic cell lines corresponded well with gene expression levels. These three proteins were CATW, MRS2, and SDCB2.

CATW (or CTSW, also known as lymphopain) is a cysteine endopeptidase belonging to the cathepsin protease superfamily [32]. CATW is predominantly expressed in natural killer (NK) cells and CD8^+^ T lymphocytes, and acts as a potential regulator of NK/T cell cytotoxicity [33]. While numerous evidence has demonstrated that cathespin family members contribute to cancer proliferation and metastasis [34], previous research has shown that CATW is a candidate tumor suppressor and is positively correlated with BC patient survival [35] (as is also validated in the current study). It has been speculated that decreased CATW expression may implicate diminished NK cell activity and lead to an increased likelihood of cancer metastasis [35]. This notion corresponds with our observation in the current dataset that exosome CATW abundance was significantly lower in all three metastatic cell lines, especially the lung metastasis site (i.e., 1424.2).

MRS2 is a magnesium transporter protein localized in the mitochondrial inner membrane, and accumulating evidence has revealed that MRS2 is essential for eukaryotic cell survival. In the context of human cancers, the dysregulation of magnesium homeostasis and the aberrant expression of magnesium transporters/channels, including MRS2, are associated with key processes of cancer metastasis, such as local invasion, transmigration, and colonization [36,37]. The up-regulation of MRS2 has also been identified in a gastric cancer cell line with a multidrug-resistant phenotype, and it may exert its effect via regulating cell cycles and releasing cytochrome C [38]. These results were in line with our observations that exosome MRS2 abundance was the highest in the primary metastatic BC cell line (i.e., 1424).

SDCB2 is a member of the PDZ protein family of systenins. Although little has been unveiled about the functions of SDCB2 in cancer, its close sibling, syntenin-1 (SDCB1), has been regarded as a key regulator of proliferation and metastasis in many cancers, including BC [39,40,41], and is one that activates various signaling cascades promoting cell proliferation and invasion, e.g., p38/NF-κB, p38/MAPK, ERK1/2, and AKT. Up-regulated SDCB1 tissue expression and serum levels were correlated with poor prognosis in lung cancer and BC patients [42]. Genetic silencing and the pharmacological inhibition of SDCB1 impeded BC cell growth by activating cytotoxic T cells and suppressed BC metastasis [43]. In our dataset, we observed that exosome SDCB2 was more abundant in the primary metastatic cell line, i.e., 1424, indicating that SDCB2 may share some functional similarities with SDCB1 and be involved in BC proliferation and metastasis.

In addition, two additional proteins also showed higher exosome abundance (0.05 < *p* < 0.1) in metastatic cell lines and higher gene expression levels in invasive BC patients, correlating with a poor prognosis. RTN4 (also known as Nogo) is a myelin-associated protein that inhibits axon outgrowth in the central nervous system, and has exhibited great potential as a therapeutic target for treating demyelinating diseases and spinal cord injuries [44,45]. Xue et al. showed that knocking down RTN4 suppressed human colorectal cancer cell growth via cell cycle arrest at the G0/G1 phase [46] and Pathak et al. reported that RTN4 knockdown decelerated cancer proliferation and enhanced paclitaxel-induced cytotoxicity mediated by AKT pathways [47]. The other marker was RAD23B, a protein involved in nucleotide excision repair (NER). Li et al. found that cytoplasmic RAD23B promoted colorectal cancer progression and metastasis by interacting with coronin 1C (CORO1C), and the silencing of RAD23B expression significantly retarded tumor growth and metastasis in vivo [48]. Other studies reported that nuclear RAD23B was a potential tumor suppressor and was decreased in the tumor tissue in women with BC [49,50]. These results suggested that RAD23B may play completely opposite roles in cancer etiology based on cellular localization.

## 4. Materials and Methods

### 4.1. Cell Culture

The EpH4-Ev, EpH4-B-MEKDD, EpH4 1424, EpH4 1424.1, and EpH 1424.2 cell lines were obtained from ATCC. Cells were plated in 15 cm Petri dishes and grown under 5% CO_2_ culture conditions at 37 °C in Dulbecco’s Modified Eagle Medium (DMEM; Corning Inc., Corning, NY, USA) supplemented with 10% exosome-free fetal bovine serum (FBS; System Biosciences, Palo Alto, CA, USA), penicillin (10 units/mL), and streptomycin (100 μg/mL) (Corning, NY, USA). When cell confluence reached 80%, cells were lysed using RIPA buffer (Boston Bio-Products; MA), supplemented with protease and phosphatase inhibitors (Thermo Scientific; MA), and Western blotting was performed as previously described (PubMed PMID: 34211974) to detect exosome markers in the whole cell lysate.

### 4.2. Purification of Epithelium-Derived Exosomes

Cell-derived exosomes were isolated via an ultracentrifugation method using a Beckman L8-70M ultracentrifuge with an SW28 rotor (Beckman Coulter, Brea, CA, USA). The cell culture supernatant was collected in 50 mL centrifuge tubes and subjected to a sequential centrifugation procedure to clean up non-exosome components in the samples: (1) 500× *g* for 10 min to remove cell debris; (2) 10,000× *g* for 30 min to remove apoptotic bodies; and (3) 50,000× *g* for 60 min to remove micro-vesicles. The supernatant was carefully transferred to new tubes between each step, without disturbing the pellet. Finally, the supernatant was centrifuged at 100,000× *g* for 90 min to harvest the exosomes. The isolated exosomes were then imaged using an imaging flow cytometry system at the RPCI core facility. A Western blot analysis of exosome markers CD64 and FLOT1 was performed on isolated exosomes and whole cell lysates using the corresponding antibodies (Boston Bio-Products, Milford, MA, USA).

### 4.3. Protein Extraction and Digestion

The isolated exosomes were re-suspended in an SC buffer (50 mM tris-formic acid pH 8.4, 150 mM NaCl, 2% SDS, 0.5% sodium deoxycholate, and 2% IGEPAL CA-630) supplemented with cOmplete protease inhibitor cocktail tablets (Roche Applied Sciences, Indianapolis, IN, USA). Re-suspended exosomes were sonicated in three sonication–cooling cycles (20 s each) and centrifuged at 18,000× *g* and 4 °C for 30 min. The supernatant was transferred to new Eppendorf tubes, and the protein concentrations for all samples were determined via bicinchoninic acid assay. For protein digestion, 50 μg protein was aliquoted from each sample and the sample volume was adjusted to 50 μL with 0.5% SDS. The protein was reduced by using 10 mM dithiothreitol (DTT) at 56 °C for 30 min and then alkylated by using 25 mM iodoacetamide (IAM) at 37 °C for 30 min in darkness. Both procedures were performed with constant shaking in a covered thermomixer (Eppendorf, Framingham, MA, USA). The protein was then precipitated via the addition of seven volumes of chilled acetone with rigorous vortexing and the mixture was incubated at −20 °C for 3 h, followed by centrifugation at 18,000× *g* and 4 °C for 30 min to pellet the protein. After removing the supernatant, the protein pellets were rinsed with 500 μL methanol, air dried for 1 min, and then wetted using 40 μL Tris-formic acid (FA) pH 8.4. A total volume of 10 μL trypsin (0.25 μg/μL, dissolved in 50 mM Tris-FA pH 8.4; Sigma-Aldrich, St. Louis, MO, USA) was added to each sample, and tryptic digestion was performed at 37 °C for 6 h with constant shaking in a covered thermomixer. Digestion was terminated by adding 0.5 μL FA, and samples were centrifuged at 18,000× *g* and 4 °C for 30 min. The supernatant was carefully transferred to LC vials for analysis.

### 4.4. Liquid Chromatography–Mass Spectrometry (LC–MS)

For each sample, peptide derived from 4 μg protein was injected for LC–MS analysis. The LC–MS system consists of a Dionex UltiMate 3000 nano LC system, a Dionex UltiMate 3000 micro LC system with a WPS-3000 autosampler, and an Orbitrap Fusion Lumos mass spectrometer (ThermoFisher Scientific, San Jose, CA, USA). The peptide was loaded onto a large inner diameter (i.d.) trapping column (300 μm i.d. × 5 mm) coupled to the analytical column (75 μm i.d. × 65 cm, packed with 2.5 μm Waters XSelect CSH C18 material). This trapping nano LC setting achieves high-capacity sample loading, online sample desalting/cleanup, and selective peptide delivery. The mobile phases A and B for nano LC were 0.1% FA in 2% acetonitrile (ACN) and 0.1% FA in 88% ACN, respectively. The nano LC gradient was 4–13% B for 5 min, 13–30% B for 117 min, 30–50% for 10 min, 50–97% B for 1 min, and isocratic at 97% B for 17 min before equilibration to 4% B. The trapping column was switched online with the nano LC column during the first 45 min of the gradient and switched offline for cleanup and equilibration. MS was operated under data-dependent acquisition (DDA) mode, with a maximal duty cycle of 3 s. The MS1 spectra were acquired via Orbitrap in an *m/z* range of 400–1500 Th under a 120k resolution with a 5 × 10^5^ automatic gain control (AGC) target, a 50 ms maximum injection time, and 45 s ± 10 ppm dynamic exclusion settings. Precursor ions were filtered by quadrupole using a 1.2-Th-wide window and fragmented via high-energy collisional dissociation (HCD) at a normalized energy of 35%. The MS2 spectra of the product ions were acquired via Orbitrap under a 15k resolution with a 4 × 10^5^ AGC target and a 50 ms maximum injection time. A detailed description of the LC–MS parameters is included in our previous publications.

### 4.5. Data Processing and Analysis

The LC–MS raw files were processed using the IonStar data processing pipeline to generate the quantification results. Protein identification was performed by matching the LC–MS raw files against the Swiss-prot *Mus musculus* protein sequence database (downloaded in June 2018, 16,961 entries) using Sequest HT embedded in Proteome Discoverer 1.4 (ThermoFisher Scientific, San Jose, CA, USA). The search parameters included (1) precursor/fragment mass tolerance: 20 ppm/0.02 Da; (2) maximal missed cleavages: 2; (3) enzyme name: trypsin (Full); (4) dynamic modifications: peptide N-terminal acetylation, methionine oxidation; and (5) static modification: cysteine carbamidomethylation. Peptide-spectrum match (PSM) filtering, protein inference/grouping, and global false discovery rate control was performed using Scaffold 4 (Proteome Software, Inc., Portland, OR, USA). The protein and peptide FDRs were set at 1, with a minimum of two unique peptides per protein. The filtered PSM list was exported and manually formatted in Excel.

Protein quantification, which encompasses quantitative feature generation and post-feature generation data processing, was performed using IonStar, an MS1 ion current-based approach with high accuracy/precision and low missing data in large sample cohorts. Major steps included: (1) chromatographic alignment using the ChromAlign algorithm to adjust inter-run retention time (RT) deviation and cluster peptide ion peaks (the selection of the optimal reference for alignment was based on the alignment scores and base peak ion chromatogram intensity); (2) data-independent MS1 peak feature (i.e., frames) generation via the Direct Ion-Current Extraction (DICE) method, which extracts ion chromatograms for all precursor ions with corresponding MS2 scans in the aligned dataset under a pre-defined m/z-RT window (10 ppm, 1 min); and (3) post-feature generation data processing using the in-house-developed R-Shiny application UHR-IonStar v1.4 (https://github.com/JunQu-Lab/UHRIonStarApp, accessed on 30 September 2022). The filtered PSM list and the quantitative feature database were first merged by an MS2 scan identifier to generate a list of annotated frames with peptide sequence assignment. The annotated frames were then subjected to dataset-wide normalization, principal-component-based detection and removal of peptide outliers, and data aggregation at the protein level. The calculation of protein ratios and statistical testing (one-way ANOVA test) was also performed by the UHR-IonStar application.

The list of *Mus musculus* and *Homo sapiens* exosome proteins were subset from two well-established extracellular vesicle/exosome databases, Vesiclepedia and ExoCarta. Statistical testing to determine altered proteins was performed by one-way ANOVA. The GO and KEGG pathway analysis was performed by the Database for Annotation, Visualization, and Integrated Discovery (DAVID) Bioinformatics Resources 6.8 (https://david.abcc.ncifcrf.gov, accessed on 5 October 2022). RPA was performed using the Reactome Pathway Database (https://reactome.org, accessed on 5 October 2022). GO terms and KEGG/Reactome pathways with *p*-values < 0.05 after the Benjamini–Hochberg procedure were exported and manually curated. The identification of associations between the identified exosome proteins and human diseases was performed by DisGeNet (https://www.disgenet.org, accessed on 8 October 2022). A protein–protein interaction network was established by STRINGDB (https://string-db.org, accessed on 27 October 2022). A survival analysis of selected proteins was accomplished using GEPIA (http://gepia.cancer-pku.cn, accessed on 17 October 2022). The plotting of figures was performed via GraphPad Prism and the ggplot2 package in R.

## 5. Conclusions

In conclusion, a quantitative proteomics study was performed to investigate the exosomes isolated from five BC cell lines from the same mammary epithelial cell lineage (N = 20 in total). IonStar, an in-house-developed proteomics approach, enabled the quantification of 2135 unique proteins with high reproducibility and zero missing data on the protein level, among which 348 proteins were determined to be significantly changed in at least one of the cell lines (i.e., altered proteins). Further informatics analysis of the altered proteins suggested the key components of exosome-mediated signaling processes involved in the etiology of BC proliferation and metastasis, such as activation of innate immunity, cell adhesion and migration, ribosomal proteins, and proteoglycans. Moreover, the expression profiles of five metastasis-specific proteins (CATW, MRS2, SDCB2, RTN4, and RAD23B) were found to be consistent with their gene expression levels in BRCA patients and correlated with the overall survival of invasive BC patients, suggesting the potential of these proteins as putative exosome markers for BC metastasis. Although there are some limitations using mouse mammary epithelial cells in the current study, the results obtained were very informative and our IonStar approach can be used as a proof of principle to investigate exosome proteomes. We plan to further carry out exosome proteomic analysis using a series of human breast cancer patient-derived cells from primary to metastatic tumors. We expect the new study to provide opportunities for novel biomarker or therapeutic target discovery for metastatic BC.

## Data Availability

The mass spectrometry proteomics data have been deposited to the ProteomeXchange Consortium via the PRIDE partner repository with the dataset identifier PXD038553. UHR-IonStar and the user manual are available at (https://github.com/JunQu-Lab/UHRIonStarApp, accessed on 30 September 2022).

## Supplementary Materials

The following supporting information can be downloaded at https://www.mdpi.com/article/10.3390/ijms24044033/s1.

## Abbreviations

BC	Breast cancer
BP	Biological process
CATW	Cathepsin W
CC	Cellular component
CV	Coefficient of variation
GEPIA	Gene Expression Profiling Interactive Analysis
GO	Gene ontology
LC	Liquid chromatography
MRS2	Magnesium transporter MRS2
MS	Mass spectrometry
PCA	Principal component analysis
PPI	Protein-protein interaction
RAD23B	UV excision repairt protein RAD23 homolog
RPA	Reactome pathway analysis
RTN4	Reticulon-4
SC	Surfactant cocktail
SDCB2	Syntenin-2
SEPOD	Surfactant cocktail-aided extraction/precipitation/on-pellet digestion
