# Supplementary material for: Comparative Proteomics Analysis of Exosomes Identifies Key Pathways and Protein Markers Related to Breast Cancer Metastasis
Source: Int J Mol Sci. 2023 Feb 17;24(4):4033. doi: 10.3390/ijms24044033 (PMC9967130; doi:10.3390/ijms24044033)
Supplement: Supplementary file 1 [file ijms-24-04033-s001.zip › Supplementary figures.pdf]

Comparative Proteomics Analysis of Exosomes Identifies Key Pathways and Protein Markers Related to Breast Cancer Metastasis

Shichen Shen [1,2,#], Chengjian Tu [1,2,#], He Shen [3], Jun Li [1,2], Costa Frangou[4], Jianmin Zhang [3,*] and Jun Qu [1,2,*]

[1] Department of Pharmaceutical Sciences, University at Buffalo, Buffalo, NY 14214, USA
[2] New York State Center of Excellence in Bioinformatics and Life Sciences, Buffalo, NY 14203, USA
[3] Department of Cancer Genetics and Genomics, Roswell Park Comprehensive Cancer Center, Buffalo, NY 14263, USA
[4] Department of Molecular and Cellular Biology, Roswell Park Comprehensive Cancer Center, Buffalo, NY 14263, USA
* Correspondence: Jianmin.Zhang@roswellpark.org (J.Z.); JunQu@buffalo.edu (J.Q.); Tel: +1-(716)845-5929 (J.Z.); +1-(716)645-4821 (J.Q.)

Supplementary Materials

- Figures S1 – S4
- Tables S1 – S8

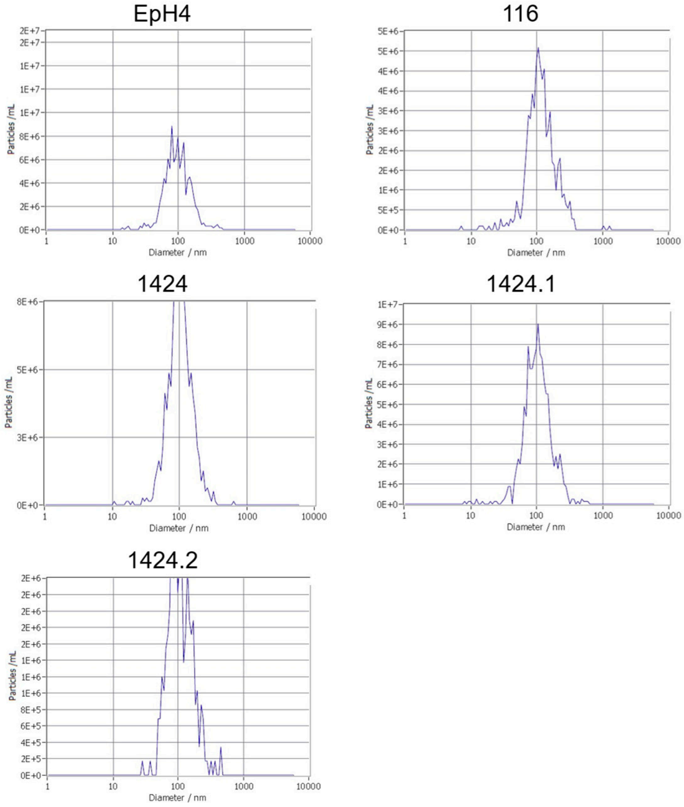

Supplementary Figure S1. Density curves (particles/mL) of exosome isolated from each EpH4 cell line.

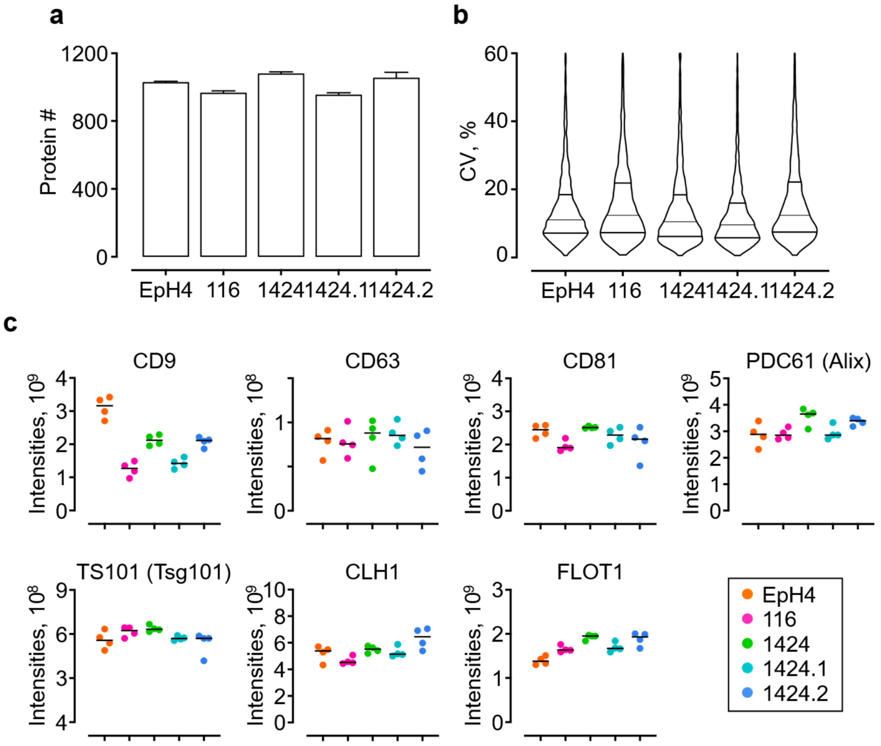

Supplementary Figure S2. Proteomics characterization of the exosomes isolated in each BC cell line. (a) the number of proteins identified; (b) Intra-group coefficient of variation (CV) of proteins; (c) MS intensities of seven canonical exosome markers.

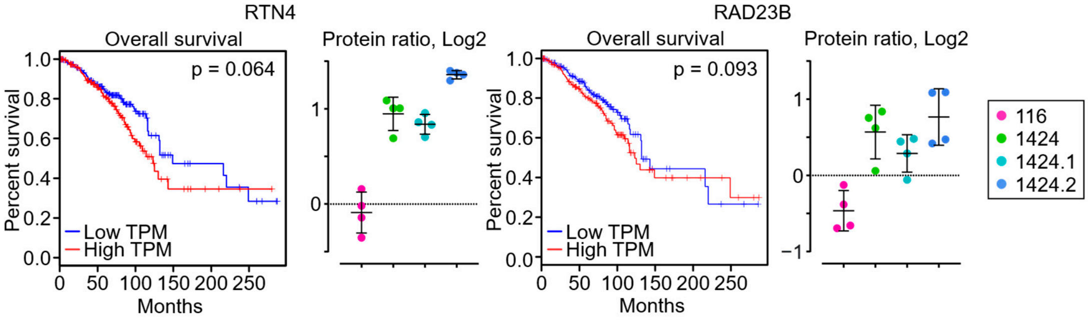

Supplementary Figure S3. Survival plots and corresponding protein exosome abundances of reticulon 4 (RTN4) and UV excision repair protein RAD23 homolog (RAD23B).

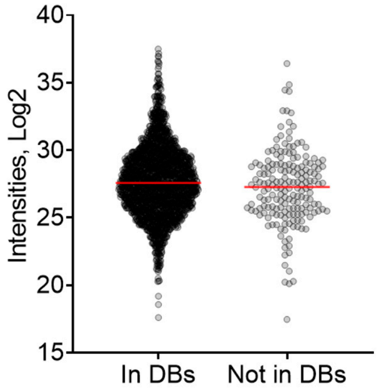

Supplementary Figure S4. MS intensities of proteins archived and not archived in the two EV databases (*i.e.*, Vesiclepedia and ExoCarta).

Table S1. List of proteins quantified and MS quantitative values in each sample.

Table S2. List of 94 quantified proteins among the TOP100 proteins in ExoCarta.

Table S3. List of protein IDs associated with BC and metastasis identified by DisGeNet.

Table S4. List of proteins with significant changes among the five cell lines (i.e., altered proteins).

Table S5. TOP20 altered proteins with the highest fold changes in each set of tumor/control comparison.

Table S6. List of altered proteins enriched in the DisGeNet terms of BC and metastasis.

Table S7. List of metastasis-specific altered proteins.

Table S8. TOP20 altered proteins with the highest fold changes in each set of metastatic/non-metastatic comparison.

**All Supplementary Tables are provided as separate Excel files.
